# A HOSPICE COLLABORATIVE NETWORK TO IMPROVE SERIOUS ILLNESS CARE IN A LARGE HEALTHCARE SYSTEM

**DOI:** 10.1093/geroni/igz038.481

**Published:** 2019-11-08

**Authors:** Richard E Leiter, Charles T Pu, Emanuele Mazzola, Rachelle E Bernacki

**Affiliations:** 1 Dana-Farber Cancer Institute, Boston, Massachusetts, United States; 2 Massachusetts General Hospital, Boston, Massachusetts, United States

## Abstract

The quality of hospice care in the United States varies significantly, yet healthcare systems lack methods to comprehensively evaluate and stimulate quality improvement in organizations that serve their patients. Partners HealthCare, an integrated healthcare system located in Eastern Massachusetts, sought to create a high-quality hospice collaborative network based on objective and quantitative criteria obtained from public reporting as well as the hospice itself. Through a modified Delphi procedure, clinicians, administrators, and data scientists developed a set of criteria and a scoring system focused on three areas: organizational information, clinical care quality indicators, and training and satisfaction. All Medicare-certified hospices in good-standing in Massachusetts were eligible to participate in a request for information (RFI) process. We blinded all hospice data prior to scoring and invited hospices scoring above the 15th percentile to join the collaborative for a 2-year initial term. Of 72 eligible hospices, the majority (53%) responded to the RFI, of which 60% submitted completed surveys. Hospices could receive up to 23.75 points with scores ranging from 2.25 to 19.5. The median score was 13.62 (IQR: 10.5-16.75). For the 19 hospices scoring above the 15th percentile, scores ranged from 10.0-19.5 (median: 14, IQR: 11.1-16.9). There was no association between quality score and continuous (Spearman’s correlation 0.24, p=0.27) or dichotomous (Wilcoxon rank sum test p=0.13) measures of hospice size. The hospice collaborative network is one healthcare system’s initial attempt to effectively leverage its influence and relationships to improve hospice quality for the benefit of its seriously ill patients and their families.

